# Effect of Transarterial Chemoembolization on ALBI Grade in Intermediate-Stage Hepatocellular Carcinoma: Criteria for Unsuitable Cases Selection

**DOI:** 10.3390/cancers13174325

**Published:** 2021-08-27

**Authors:** Chen-Ta Chi, I-Cheng Lee, Rheun-Chuan Lee, Ya-Wen Hung, Chien-Wei Su, Ming-Chih Hou, Yee Chao, Yi-Hsiang Huang

**Affiliations:** 1 Division of Gastroenterology and Hepatology, Department of Medicine, Taipei Veterans General Hospital, Taipei 11217, Taiwan; ctchi2@vghtpe.gov.tw (C.-T.C.); iclee@vghtpe.gov.tw (I.-C.L.); ywhung2@vghtpe.gov.tw (Y.-W.H.); cwsu2@vghtpe.gov.tw (C.-W.S.); mchou@vghtpe.gov.tw (M.-C.H.); 2 Institute of Clinical Medicine, School of Medicine, National Yang Ming Chiao Tung University, Taipei 11221, Taiwan; 3 Department of Radiology, Taipei Veterans General Hospital, Taipei 11217, Taiwan; rclee@vghtpe.gov.tw; 4 Department of Oncology, Taipei Veterans General Hospital, Taipei 11217, Taiwan; ychao@vghtpe.gov.tw

**Keywords:** hepatocellular carcinoma, HBV, transarterial chemoembolization, tumor burden, ALBI-grade migration

## Abstract

**Simple Summary:**

BCLC-B HCC encompasses heterogeneous populations with varied tumor burden and liver reserve resulting in diverse clinical outcomes to TACE. Liver function deterioration would happen after TACE in patients with high tumor burden. Here, we found that the risk of post-TACE acute ALBI-grade migration was 24.3% and chronic ALBI-grade migration was 16% for BCLC-B HCC patients; HBV infection, up-to-seven criteria, and up-to-eleven criteria were factors of acute ALBI-grade migration, whereas bilobar tumor involvement had high risk of chronic ALBI migration once acute ALBI-grade migration developed after TACE. Overall, up-to-eleven criteria consistently associated with acute and chronic ALBI-grade migration, suggesting that up-to-eleven is an appropriate parameter to select TACE-unsuitable HCC patients who are at risk of liver function deterioration. In addition, patients with ALBI-grade migration in acute or chronic phases had significantly poorer PFS than patients without ALBI-grade migration.

**Abstract:**

Transarterial chemoembolization (TACE) is the standard of care for intermediate stage hepatocellular carcinoma (HCC). We aimed to identify unsuitable cases who were at risk of ALBI-grade migration by TACE. Consecutive 531 BCLC-B HCC patients undergoing TACE were reviewed, and factors associated with ALBI-grade migration were analyzed. There were 129 (24.3%) patients experienced acute ALBI-grade migration after TACE, and 85 (65.9%) out of the 129 patients had chronic ALBI-grade migration. Incidences of acute ALBI-grade migration were 13.9%, 29.0% for patients within or beyond up-to-7 criteria (*p* < 0.001) and 20.0%, 36.2% for patients within or beyond up-to-11 criteria (*p* < 0.001), respectively. HBV infection, tumor size plus tumor number criteria were risk factors associated with acute ALBI-grade migration. Bilobar tumor involvement was the risk factor of chronic ALBI-grade migration in patients with acute ALBI-grade migration. Up-to-eleven (*p* = 0.007) performed better than up-to-seven (*p* = 0.146) to differentiate risk of dynamic ALBI score changes. Moreover, ALBI-grade migration to grade 3 has adverse effect on survival. In conclusion, tumor burden beyond up-to-eleven was associated with ALBI-grade migration after TACE, indicating that up-to-eleven can select TACE-unsuitable HCC patients who are at risk of liver function deterioration.

## 1. Introduction

Hepatocellular carcinoma (HCC) is the sixth most common malignancy and the fourth leading cause of cancer-related death worldwide [1]. Currently, Barcelona Clinic Liver Cancer (BCLC) is the most widely accepted staging system with a linkage to treatment and being recommended by the American Association for the Study of Liver Diseases (AASLD) and the European Association for the Study of the Liver (EASL) [2,3]. Transarterial chemoembolization (TACE) is the standard of care for BCLC-B, intermediate-stage HCC [4,5,6,7,8]. Although TACE can provide survival benefit to BCLC-B HCC in previous studies, liver function deterioration would happen after the procedure in patients with high tumor burden [9]. In general, BCLC-B HCC encompasses heterogeneous populations with varied tumor burden and liver reserve resulting in diverse clinical outcomes to TACE [3,10,11,12]. The concept of TACE unsuitable is proposed recently, as of the evidence that not all BCLC-B HCC patients are candidates for TACE [13].

BCLC-B subclassification had been initially proposed by Bolondi et al. [10] based on Child–Pugh score and Up-To-Seven criteria [14] to identify TACE unsuitable cases. Although this subclassification was validated in untreated HCC patients showing a significantly different survival between contiguous stages [15]. Subsequent studies struggled to reproduce its advantage in differentiating survivals among subgroups [16,17,18,19]. Therefore, several subclassifications or prognostic scores, including Kinki criteria, up-to-eleven, six-and-twelve score, were proposed to discriminate survivals of BCLC-B HCC patients after TACE [18,19,20,21,22]. Albumin-Bilirubin (ALBI) is a useful marker, simplifying from the items of Child–Pugh class, to assess liver function across different stages of HCC [23]. ALBI grade has been reported as a key factor associated with overall survival for patient on sorafenib or immunotherapy for HCC [24,25]. Decline in liver function after TACE is inevitable especially in patients with high tumor burden, which may offset the survival benefit provided by TACE [12,26,27,28]. Based on different definitions of hepatic failure, the incidence of post-TACE decompensation ranged from 5% to 49% [12,26,29,30,31]. However, the effect of TACE on ALBI, and the optimal cut-off of tumor burden to differentiate the risk of ALBI score change including ALBI-grade migration has not been well evaluated.

In this study, we tried to identify factors associated with TACE-related ALBI-grade migration in acute and chronic phases and delineate the performance of previous reported BCLC-B subclassifications in selecting high risk group for TACE.

## 2. Materials and Methods

### 2.1. Study Design and Patient Population

From October 2007 to January 2017, consecutive 531 treatment-naïve BCLC-B HCC patients undergoing TACE as the initial treatment with evaluable image studies in Taipei Veterans General Hospital were retrospectively reviewed. The diagnosis of HCC was based on the AASLD guidelines [2]. The indications of TACE had been discussed in multidisciplinary meeting, composed of interventional radiologists, gastroenterologists, hepatic surgeons, radio-oncologists, and medical oncologists.

Patients’ medical history and tumor characteristics from images were carefully recorded. The laboratory data included complete blood count, liver function test, coagulation test, hepatitis B virus (HBV), and hepatitis C virus (HCV) markers, and serum alpha-fetoprotein (AFP) levels. The baseline serum biochemistry was determined according to the most recent measurements before the TACE. Liver function was assessed by Child–Pugh scores and ALBI grade [23,32]. Incidences of TACE-related ALBI-grade migration in acute and chronic phases, progression-free survival (PFS), and overall survival (OS) were investigated. This study was approved by the Institutional Review Board, Taipei Veterans General Hospital. The study was conducted according to the principles in the Declaration of Helsinki 2013.

### 2.2. Transarterial Chemoembolization

At first, tumor stains and tumor feeding artery were identified, then catheterization was superselectively advanced into the branches of tumor feeding artery with a 1.98-/2.5-Fr microcatheter through a 4-/5-Fr catheter (Terumo, Tokyo, Japan or Cook Medical, Bloomington, IN, USA). The subsegmental TACE was performed with a mixture of 20–30 mg adriamycin (Carlo Erba, Milan, Italy) and 5–10 mL of lipiodol (Laboratoire Guerbet, Paris, France), followed by the delivery of 2–3 mm^2^ strips of Gelfoam (Upjohn Co., Kalamazoo, MI, USA). Based on the tumor size and baseline liver function, interventional radiologist determined the total amount of iodized oil individually. During procedure, all target tumors were assessed by decreased antegrade tumor-feeding arterial flow. The embolization endpoint was reduced or no tumor stain along with subjective angiographic chemoembolization endpoint levels 2 and 3 [33].

### 2.3. Definitions

Up-to-X criteria was based on the sum of the size of the largest tumor (in cm) plus the number of tumors. Acute ALBI-grade migration was defined as deterioration of ALBI from grade 1 to grade 2/3 or from grade 2 to grade 3 within 1 month after the TACE. Chronic ALBI-grade migration was defined as the events of ALBI-grade migration lasted for more than 1 month after the TACE. HBV reactivation was defined as a 10-fold increase in HBV DNA from baseline, reappearance of HBsAg in HBsAg-negative case, HBV DNA from undetectable to higher than 1000 IU/mL, or HBV DNA higher than 10,000 IU/mL if the baseline level is not available [34,35,36]. HCV reactivation was defined as an increase in HCV-RNA ≥1 log_10_IU/mL over baseline, and hepatitis flare was determined by an increase in alanine aminotransferase (ALT) to ≥3 times the upper limit of normal [37]. Liver cirrhosis was diagnosed either by abdominal sonography or CT/MRI imaging studies. Disease progression was defined by unTACEable progression as previously reported [38]. PFS was defined as the time from the date of initial diagnosis of HCC to disease progression or death, and OS was measured from the date of initial diagnosis of HCC to the date of death or the last follow-up.

### 2.4. Follow-Up and Outcomes

Liver function including Child–Pugh score was evaluated within 2 days after the TACE, then 2 weeks after TACE, and followed by every 1 to 3 months thereafter. In the presence of ALT flare up to 5 time of upper limit normal (ULN) or liver decompensation (T bilirubin ≥ 2 mg/dL), a weekly monitoring was performed. All patients had followed-up dynamic computed tomography (CT) or magnetic resonance imaging (MRI) of the liver one month after the TACE. If residual viable tumors were confirmed by dynamic CT or MRI studies, on demand TACE would be performed. If no residual tumor was identified, then abdominal sonography was performed at 3-month intervals thereafter, repeated CT or MRI was arranged in the suspicion of recurrent tumors.

### 2.5. Statistical Analysis

Continuous variables were expressed as mean with standard deviation or median with range, whereas categorical data were presented as number with percentage. The chi-squared test or Fisher exact test was performed for categorical data comparison. The Kaplan–Meier method was used for the survival curves, and the log-rank test was used to assess the differences in survival. Logistic regression model was used to identify risk factors of acute and chronic ALBI-grade migration after TACE, and odds ratio (OR) and confidence interval (CI) were evaluated. Cox proportional hazard regression analyses was used to identify risk factors for PFS and OS. Continuous variables were categorized, and variables with *p* values < 0.10 in univariate analyses were included in the final multivariate models with backward method (*p* for removal > 0.05). Highly correlated variables were not used together in the LR model in order to avoid collinearity. Therefore, models with different cut-off values of up-to-X score were evaluated separately in multivariate analysis. We used a linear mixed model with random effects on the intercept and slope of ALBI score. Meanwhile, we assessed the slope coefficient differences in patients within or beyond up-to score according to different up-to-X criteria. All tests for differences were two-tailed, and *p* values < 0.05 were considered statistically significant. All statistical analyses were performed using the Statistical Package for Social Sciences (SPSS 26.0 for Windows, SPSS Inc, Chicago, IL, USA).

## 3. Results

### 3.1. Basic Characteristics of the BCLC-B HCC Patients

Table 1 lists the demographic and baseline characteristics of the 531 BCLC-B HCC patients undergoing TACE. The mean age was 69 years old; male gender was predominant. Main etiologies of underlying liver disease were hepatitis B virus (HBV) infection (46.0%), hepatitis C virus (HCV) infection (33.9%), and alcoholism (12.4%). Most patients were in Child–Pugh class A, and ALBI grade 1/2. The mean tumor size was 6.58 cm, 48.6% of the patients had tumors involving both lobes of the liver. There were 83 patients (15.6%) within up-to-6 criteria; 165 patients (31.1%) within up-to-7 criteria; 390 patients (73.4%) within up-to-11 criteria; 424 patients (79.8%) within up-to-12 criteria, respectively. The mean sessions of TACE were 3.3 (median: 2.0; range: 1–15) per patient in the study cohort.

### 3.2. Incidence of Acute and Chronic ALBI-Grade Migration after TACE

The case numbers with ALBI-grade migration in acute and chronic phases after TACE were listed in Table 2. There were 129 (24.3%) patients that experienced ALBI-grade migration in acute phase, including 86 patients from ALBI grade 1 to ALBI grade 2, three patients from ALBI grade 1 to grade 3, and 40 patients from ALBI grade 2 to grade 3. Finally, 85 (65.9%) out of the 129 patients with ALBI-grade migration in acute phase had chronic ALBI-grade migration, including 64 patients migrating from ALBI grade 1 to grade 2, and 21 patients from ALBI grade 2 to grade 3.

### 3.3. Distribution of ALBI Grade before and after TACE in Acute Phase by Different Tumor Size plus Tumor Number Criteria

**Figure 1:**
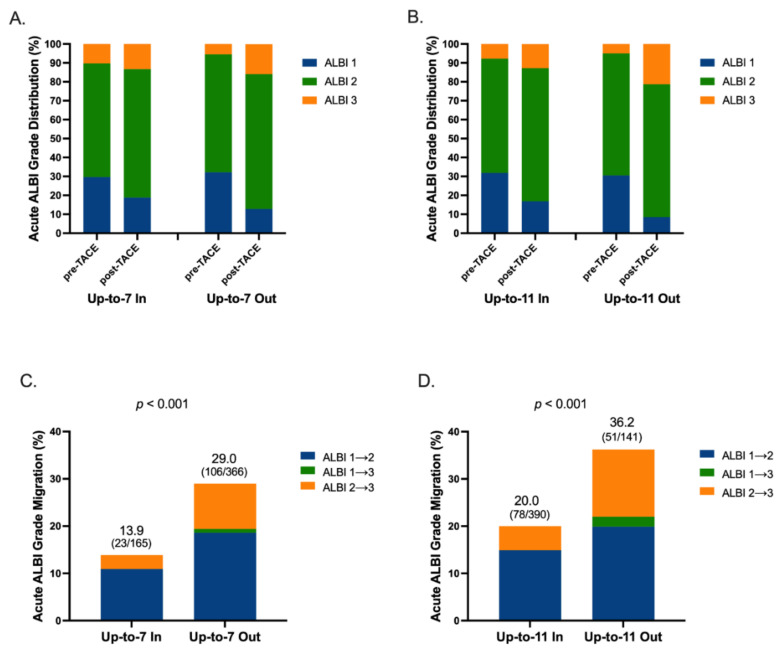
Distribution of ALBI grade and rates of acute ALBI migration. Distribution of ALBI grade stratified by (**A**) up-to-7 and (**B**) up-to-11, and acute ALBI-grade migration rate stratified by (**C**) up-to-7 and (**D**) up-to-11. Abbreviations: ALBI, Albumin-Bilirubin; TACE, transarterial chemoembolization.

The distribution of ALBI grade before and after TACE by different tumor size plus tumor number criteria in acute phase is illustrated in Figure 1A,B and Figure S1A,B. The incidences of acute ALBI-grade migration were 15.7% and 25.9% in patients within or beyond up-to-six criteria (*p* = 0.063; Figure S1C), 13.9% and 29.0% in patients within or beyond up-to-seven criteria (*p* < 0.001; Figure 1C), 20.0% and 36.2% in patients within or beyond up-to-eleven criteria (*p* < 0.001; Figure 1D), and 21.7% and 34.6% in patients within or beyond up-to-twelve criteria, respectively (*p* = 0.008; Figure S1D). Note that all the patients with ALBI migration from grade 1 to 3 were allocated in tumor size plus tumor number criteria out subgroups.

### 3.4. Factors Associated with ALBI-Grade Migration in Acute Phase

Univariate and multivariate analyses of factors associated with acute ALBI-grade migration are shown in Table 3. In univariate analysis, HBV infection, high platelet count, large tumor size, beyond up-to-X criteria, and high alpha-fetoprotein (AFP) level were associated with acute ALBI-grade migration. In multivariate analysis, HBV infection, beyond up-to-seven, or up-to-eleven criteria were independent risk factors associated with acute ALBI-grade migration in individual model.

### 3.5. Incidence of HBV Reactivation and HCV Hepatitis Flare after TACE

As HBV-HCC cases had higher risk of ALBI-grade migration in the acute phase, the risk of HBV reactivation after TACE was further delineated. Of the 244 HBV-HCC patients, 69 (28.3%) patients had on nucleos(t)ide analogs (NUCs) therapy before TACE. The incidence of ALBI-grade migration in acute phase was similar between patients with or without NUCs treatment (31.9% in patients with NUCs vs. 28.0% in patients without NUCs, *p* = 0.536; Table S1). There were eight patients that experienced HBV reactivation. The incidence of HBV reactivation after TACE were 6.1% (3/49) in patients with acute ALBI-grade migration vs. 4.0% (5/126) in patients without acute ALBI-grade migration, respectively (*p* = 0.688).

Of the 180 HCV-HCC patients, 162 patients had either prior documented antiviral therapy or undetectable HCV-RNA before TACE. Only 18 (10.6%) patients with detectable HCV-RNA before the TACE. The median HCV-RNA was 102,650 IU/mL (ranged, 92.5–7,150,000 IU/mL). Of them, no patient had experienced ALT flare within 1 month after the TACE.

### 3.6. Factors of Chronic ALBI-Grade Migration in Patients with Acute ALBI Migration after TACE

**Figure 2:**
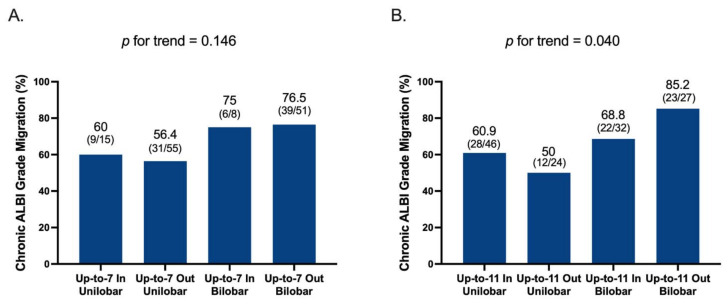
Incidence of chronic ALBI-grade migration stratified by different up-to-score plus bilobar tumor location model. (**A**) Up-to-7 plus bilobar tumor location model, and (**B**) up-to-11 plus bilobar tumor location model. Abbreviations: ALBI, Albumin-Bilirubin; TACE, transarterial chemoembolization.

Of the 129 patients with acute ALBI-grade migration, 85 (65.9%) could not recover their liver reserve. Factors associated with chronic ALBI-grade migration after acute ALBI migration are shown in Table 4. In univariate analysis, bilobar tumor involvement (OR, 2.411; *p* = 0.024; 95% CI, 1.123–5.176) was the only prognostic factor of chronic ALBI-grade migration. Figure 2 illustrated incidences of chronic ALBI migration stratified by up-to-seven and up-to-eleven with or without bilobar tumor involvement. Of them, tumor burden beyond up-to-eleven and bilobar tumor involvement had the highest risk (85.2%) of chronic ALBI-grade migration. Up-to-eleven performed better than up-to-seven in combination with bilobar tumor involvement to differentiate the risk of chronic ALBI migration among patients with ALBI changes in acute phase (*p* = 0.040; Figure 2B).

### 3.7. Dynamic Changes of ALBI Score after TACE

**Figure 3:**
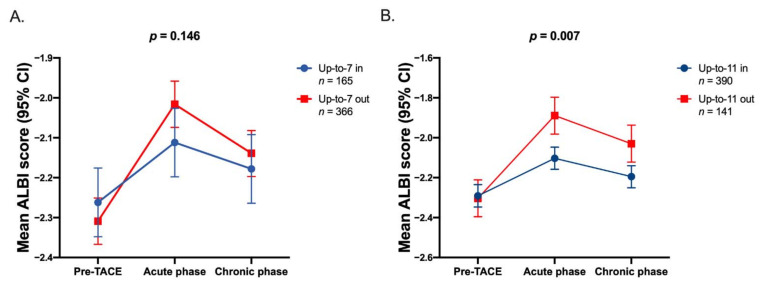
Dynamics of ALBI score in pre-TACE phase, acute phase, and chronic phase, stratified by different tumor numbers plus tumor size score. (**A**) Up-to-7 and (**B**) up-to-11. Abbreviations: ALBI, Albumin-Bilirubin; TACE, transarterial chemoembolization; CI, confidence interval.

Figure 3 showed dynamic ALBI score changes from pre-TACE phase, acute phase, and chronic phase, stratified by different tumor size plus tumor number criteria in 531 HCC patients. There was no difference in ALBI score between up-to-seven in or out (Figure 3A) patients throughout the TACE course. Importantly, up-to-eleven (*p* = 0.007) performed better to differentiate the risk of ALBI score changes both in acute and chronic phases after TACE (Figure 3B).

### 3.8. Progression-Free and Overall Survival Stratified by ALBI-Grade Migration

**Figure 4:**
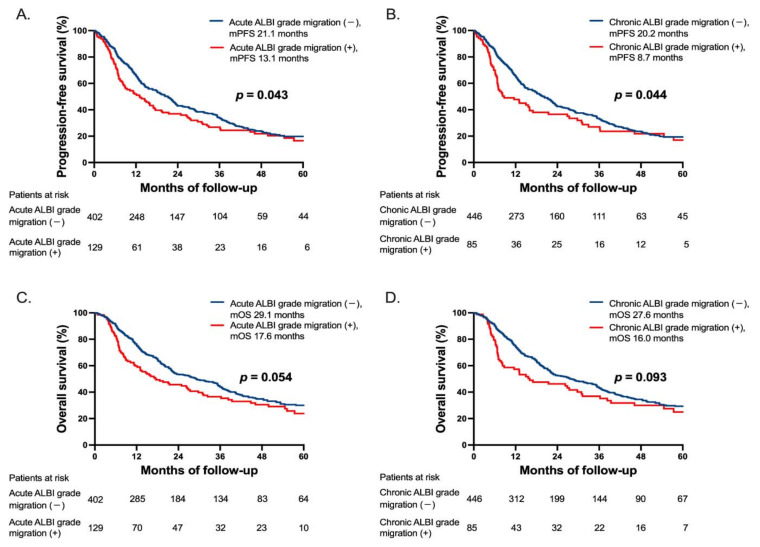
Progression-free survival (PFS) and overall survival (OS) in patients with or without ALBI-grade migration. (**A**) PFS between patients with or without acute ALBI-grade migration. (**B**) PFS between patients with or without chronic ALBI-grade migration. (**C**) OS between patients with or without acute ALBI-grade migration and (**D**) OS between patients with or without chronic ALBI-grade migration. Abbreviations: ALBI, Albumin-Bilirubin; TACE, transarterial chemoembolization; mPFS, median progression-free survival; mOS, median overall survival.

During the median follow-up period of 19.7 months (range: 1.4–111.7 months), 113 (21.3%) patients developed disease progression and 344 (64.8%) deaths occurred, including seven patients died within 1 month after the TACE. The median progression-free survival (PFS) was 13.1 months in patients with acute ALBI-grade migration after TACE vs. 21.1 months without acute ALBI-grade migration (*p* = 0.043; Figure 4A) and 8.7 months vs. 20.2 months in patients with or without chronic ALBI-grade migration, respectively (*p* = 0.044; Figure 4B). The median overall survival (OS) was 17.6 months in patients with acute ALBI-grade migration after TACE vs. 29.1 months without acute ALBI-grade migration (*p* = 0.054; Figure 4C); and 16.0 months vs. 27.6 months in patients with or without chronic ALBI-grade migration, respectively (*p* = 0.093; Figure 4D).

We further divided ALBI-grade migration into “migration to grade 2” and “migration to grade 3” (Figure S3). The median PFS was 19.3 months versus 8.2 months in patients with migration to grade 2 versus grade 3 in acute phase (*p* < 0.001; Figure S3A), and it was 13.1 months versus 5.7 months in patients with chronic ALBI migration to grade 2 versus grade 3 (*p* = 0.005; Figure S3B). The median OS was 30.9 months versus 8.9 months in patients with acute ALBI-grade migration to grade 2 versus grade 3 (*p* < 0.001; Figure S3C); and was 30.9 months versus 5.7 months in patients with chronic ALBI-grade migration to grade 2 versus grade 3 (*p* < 0.001; Figure S3D), respectively.

### 3.9. Prognostic Factors Associated with PFS and OS

Univariate and multivariate analyses of factors associated with PFS were shown in Table S2. In univariate analysis, large tumor size, high AFP level, and ALBI-grade migration to grade 3 in acute or chronic phase after TACE were associated with PFS. In multivariate analysis, tumor size, AFP > 400 ng/mL, and ALBI-grade migration to grade 3 were independent risk factors associated with poor PFS in individual model. For OS (Table S3), high aspartate aminotransferase (AST) level, large tumor size, high AFP level, and ALBI-grade migration to grade 3 were risk factors in univariate analysis. In multivariate analysis, age > 70, AST > 45 IU/L, large tumor size, AFP > 400 ng/mL, and ALBI-grade migration to grade 3 were independent risk factors associated with OS in individual model.

## 4. Discussion

To the best of our knowledge, this is the first study to investigate the impact of TACE on acute and chronic ALBI-grade migration for BCLC-B HCC patients and their risk factors. The main findings of the present study included that the risk of post-TACE acute ALBI-grade migration was 24.3%, chronic ALBI-grade migration was 16% (accounting for 65.9% of the acute ALBI-grade migration cases) for BCLC-B HCC patients; HBV infection, up-to-seven criteria and up-to-eleven criteria were factors of acute ALBI-grade migration, whereas bilobar tumor involvement had high risk of chronic ALBI migration once acute ALBI-grade migration developed after TACE. Overall, up-to-eleven criteria consistently associated with acute and chronic ALBI-grade migration, which can identify untoward ALBI score changes. Patients with ALBI-grade migration to grade 3 had an adverse effect on survival.

ALBI grade by eliminating subjective variables among Child–Pugh score, was recently developed to assess liver reserve in HCC patients [23]. More recent studies have validated the prognostic ability of ALBI grade for HCC patients undergoing TACE [21,39,40]. Previous studies reported that liver decompensation after TACE is more frequent in patients with poor liver reserve and higher tumor burden [29,30,41,42,43]. However, the effect of TACE on dynamic change of ALBI grade and the risk factors of ALBI-grade migration by TACE have not been reported yet. In our study, we evaluated liver function deterioration after one session of TACE to eliminate the confounding effect of multiple sessions of TACE on hepatic reserve, which was consistent with most of the previous TACE studies [29,30,42,43].

HBV reactivation can occur following TACE and radiofrequency ablation (RFA) for HCC, which may lead to liver function deterioration [44,45,46]. One randomized controlled study demonstrated that preemptive lamivudine therapy can reduce hepatitis due to HBV reactivation and hepatic morbidity during transarterial chemo-lipiodolization (TACL) [47]. Furthermore, one study found that patients without antiviral treatment had higher rates of HBV reactivation and liver function deterioration in TACE group compared with hepatectomy group, and HBV reactivation was a predictive factor of liver function exacerbation after TACE [48]. However, the risk of HBV exacerbation related to TACE was reported to be low [49]. In our study, the incidence of acute and chronic ALBI migration between patients with or without NUCs was comparable. The overall HBV reactivation rate after TACE was 4.6% (8/175) in patients without NUCs. We also observed that the risk of HBV reactivation was similar between patients with or without acute ALBI-grade migration. Note that the mean tumor size was significantly larger in HBV-HCC than in HCV-HCC (7.05 ± 3.92 cm vs. 5.47 ± 2.93 cm, *p* < 0.001, Table 1), which might partly explain HBV as a risk factor of acute ALBI migration after TACE.

In Table 2, of the 167 patients with ALBI grade 1 at baseline, 89 had ALBI-grade migration to ALBI 2 or 3, while among the 327 patients with ALBI grade 2 at baseline, only 40 had ALBI-grade migration to ALBI grade 3 at acute phase. It is interesting to know why ALBI grade 1 patients had higher risk of ALBI-grade migration after TACE. Among the 167 patients with ALBI grade 1 before TACE, 49 (29.3%) were within up-to-7, 75 (44.9%) were intermediate tumor burden (7–11), and 43 (25.7%) were beyond up-to-11, respectively. For patients with ALBI grade 2/3, 116 (31.9%) were within up-to-7, 150 (41.2%) were between 7 to11, and 98 (26.9%) patients were beyond up-to-11, respectively. The distributions of tumor burden were of no difference between ALBI grade 1 versus ALBI grade 2/3 patients (*p* = 0.718). Of noted, ALBI grade 1 intermediate stage HCC patients were majorly located near the cut-off of ALBI 1 and 2 (median ALBI score was −2.83; ranged from −3.53 to −2.60), consequently those cases would be liable to become ALBI grade 2 after TACE.

Considering ALBI migration deteriorated unidirectionally, and ALBI-grade migration could happen in most ALBI grade 1 and few ALBI grade 2 patients. Consequently, patients with preserved baseline liver function seemed to be at higher risk of ALBI grade change after TACE. Therefore, no parameter of liver function reserve could be identified as factor of ALBI-grade migration.

There are several definitions of high tumor burden for intermediate stage HCC, including up-to-seven (Bolondi’s sub-classifications) [10], Kinki criteria [18], and STATE score [50]. Our recent ALBI-TAE model suggested that up-to-eleven criteria is more discriminative than up-to-seven criteria to predict survival after TACE [19,21], whereas six-twelve criteria can stratify recommended TACE candidates [22]. Most recently, we proposed seven-eleven criteria to redefine tumor burden, which can predict radiologic response and survival in HCC patients undergoing TACE [33]. Taking together, high tumor burden is agreed to be an important parameter to select TACE unsuitable patients [13,51,52]. We further apply seven-eleven criteria to determine its performance in ALBI score changes by TACE (Figure S2). Patients with high tumor burden (beyond up-to-11) had the greatest changes in ALBI score among three groups (*p* for trend = 0.033), which suggesting that up-to-eleven is an appropriate parameter to select TACE-unsuitable HCC patients.

ALBI-grade migration after the first time TACE implying worse PFS in our data. We adopted unTACEable as the definition of progression in this study. Our finding confirmed the concept of TACE unsuitable [13] that TACE for cases with high tumor burden might result in ALBI-grade migration, and subsequently be liable to become unTACEable. Although the effect of ALBI migration on OS was marginal, this might be related to multiple systemic therapies emerging in these years which might prolong the survival after disease progression. In multivariate analysis, tumor burden including tumor size and high AFP level determined the PFS and OS. Because both acute and chronic ALBI-grade migrations are highly associated with tumor burden, this finding still implies the importance of ALBI migration in outcomes of TACE.

This study has some limitations. First, it was a single-center, retrospective study. However, the sample size and the follow-up time should be large and long enough. Second, HBV and HCV are key etiology of HCC in Taiwan, and it might present different tumor characteristics from other regions where HCC is related to non-alcoholic steatohepatitis. Third, there were lack of serial HCV viral loads after the TACE for the 18 HCV viremic HCC patients. However, HCV-HCC was not the risk factor of ALBI-grade migration in uni- and multivariate analyses. Fourth, the technique of TACE is highly associated with the changes of ALBI grade but is also operator-dependent. Nevertheless, our interventional radiologists were well trained and highly experienced with TACE and would try their best to do a superselective catheterization during the procedure. Last, not all BCLC-B subclassifications for TACE were evaluated in our study.

## 5. Conclusions

This study confirms that tumor burden beyond up-to-eleven was associated with ALBI-grade migration after TACE. Up-to-eleven criteria may be applied in clinical practice to identify high risk group of TACE.

## Figures and Tables

**Table 1:** Basic characteristics of BCLC-B HCC patients undergoing TACE treatment (*n* = 531).

Basic Characteristics	BCLC-B HCC
	*n* = 531
Age (years), mean ± S.D.	69.0 ± 12.3
Gender, Male, *n* (%)	412 (77.6)
HBsAg, Positive, *n* (%)	244 (46.0)
Anti-HCV, Positive, *n* (%)	180 (33.9)
Alcoholic, *n* (%)	66 (12.4)
Cirrhosis, *n* (%)	379 (71.4)
Platelet count (×10^4^/μL), median (range)	141 (22–601)
ALT (IU/L), median (range)	46 (7–355)
AST (IU/L) ^†^, median (range)	54 (6–806)
INR, median (range)	1.07 (0.85–14.50)
Albumin (g/dL), median (range)	3.6 (2.1–4.9)
Total bilirubin (mg/dL), median (range)	0.77 (0.19–3.79)
Child-Pugh class, A/B, *n* (%)	459 (86.4)/72 (13.6)
ALBI grade, 1/2/3, *n* (%)	167/327/37 (31.5/61.6/7.0)
Tumor size (cm), mean ± SD	6.58 ± 3.72
HBV-HCC (*n* = 220)	7.05 ± 3.92
HCV-HCC (*n* = 156)	5.47 ± 2.93
HBV-HCV-HCC (*n* = 24)	6.21 ± 3.21
Tumor location, Unilobar/Bilobar, *n* (%)	273 (51.4)/258 (48.6)
Tumor number, ≥3/<3, *n* (%)	269 (50.7)/262 (49.3)
Tumor size plus tumor number models	
Up-to-6, In/Out, *n* (%)	83/448 (15.6/84.4)
Up-to-7, In/Out, *n* (%)	165/366 (31.1/68.9)
Up-to-11, In/Out, *n* (%)	390/141 (73.4/26.6)
Up-to-12, In/Out, *n* (%)	424/107 (79.8/20.2)
AFP (ng/mL), median (range)	50.33 (1.00–1,050,960.00)

^†^ One missing data; Abbreviations: BCLC, Barcelona Clinic Liver Cancer; HCC, hepatocellular carcinoma; TACE, transarterial chemoembolization; S.D., standard deviation; HBsAg, hepatitis B surface antigen; HCV, hepatitis C virus; ALT, alanine transaminase; IU, international unit; AST, aspartate aminotransferase; INR, international normalized ratio; ALBI, Albumin-Bilirubin; AFP, alpha-fetoprotein.

**Table 2:** ALBI grade changes in acute and chronic phases after TACE treatment (*n* = 531).

Post-TACE ALBI Grade		Pre-TACE ALBI Grade		
		ALBI Grade 1	ALBI Grade 2	ALBI Grade 3
Acute phase	ALBI Grade 1	78 (14.7%)	0	0
	ALBI Grade 2	86 (16.2%)	287 (54.0%)	0
	ALBI Grade 3	3 (0.6%)	40 (7.5%)	37 (7.0%)
Chronic phase	ALBI Grade 1	103 (19.4%)	0	0
	ALBI Grade 2	64 (12.1%)	306 (57.6%)	0
	ALBI Grade 3	0	21 (3.9%)	37 (7.0%)

Abbreviations: ALBI, Albumin-Bilirubin; TACE, transarterial chemoembolization.

**Table 3:** Factors associated with acute ALBI-grade migration of the 531 BCLC-B HCC patients undergoing TACE.

Factors	No.	Univariate		Multivariate							
				Model 1		Model 2		Model 3		Model 4	
		OR (95% CI)	*p*	OR (95% CI)	*p*	OR (95% CI)	*p*	OR (95% CI)	*p*	OR (95% CI)	*p*
Age (years), >70/≤70	265/266	1.209 (0.812–1.798)	0.350	-							
Gender, Male/Female	412/119	1.446 (0.872–2.398)	0.153	-							
HBV vs. HCV ^†^	220/156	2.092 (1.263–3.465)	0.004	2.046 (1.232–3.399)	0.006	1.968 (1.178–3.288)	0.010	1.882 (1.125–3.146)	0.016	2.061 (1.243–3.416)	0.005
Alcoholic, Yes/No	66/465	1.196 (0.668–2.141)	0.547	-							
Cirrhosis, Yes/No	379/152	0.710 (0.464–1.086)	0.114	-							
PLT (K), ≤120/>120	196/335	0.557 (0.360–0.862)	0.009	0.830 (0.491–1.403)	0.487	0.911 (0.533–1.557)	0.733	0.883 (0.516–1.511)	0.650	0.848 (0.498–1.446)	0.545
ALT (IU/L), >40/≤40	306/225	1.218 (0.812–1.827)	0.340	-							
AST (IU/L) ^‡^, >45/≤45	325/205	1.462 (0.960–2.228)	0.077	1.302 (0.774–2.190)	0.320	1.230 (0.728–2.077)	0.439	1.209 (0.710–2.056)	0.485	1.271 (0.751–2.151)	0.372
INR, >1.0/≤1.0	427/104	1.440 (0.844–2.456)	0.181	-							
Tumor location Bilobar/Unilobar	258/273	0.860 (0.578–1.280)	0.457	-							
Tumor size, per cm	531	1.133 (1.075–1.193)	<0.001	-							
Tumor number, ≥3/<3	269/262	0.739 (0.497–1.101)	0.138	-							
Up-to-6, Out/In	448/83	1.881 (1.003–3.527)	0.049	2.031 (0.952–4.330)	0.067	-	-	-	-	-	-
Up-to-7, Out/In	366/165	2.517 (1.534–4.129)	<0.001	-	-	2.838 (1.567–5.141)	0.001	-	-	-	-
Up-to-11, Out/In	141/390	2.267 (1.484–3.463)	<0.001	-	-	-	-	1.836 (1.095–3.080)	0.021	-	-
Up-to-12, Out/In	107/424	1.907 (1.204–3.023)	0.006	-	-	-	-	-	-	1.625 (0.919–2.875)	0.095
AFP (ng/mL), >400/≤400	149/382	1.686 (1.104–2.574)	0.016	1.260 (0.756–2.100)	0.376	1.181 (0.705–1.979)	0.528	1.266 (0.759–2.111)	0.367	1.316 (0.792–2.185)	0.289

^†^ exclude 24 co-infection patients; ^‡^ One missing data; Abbreviations: ALBI, Albumin-Bilirubin; BCLC, Barcelona Clinic Liver Cancer; HCC, hepatocellular carcinoma; TACE, transarterial chemoembolization; OR, odds ratio; CI, confidence interval; HBV, hepatitis B virus; HCV, hepatitis C virus; PLT, platelet count; K, kilo; ALT, alanine transaminase; AST, aspartate aminotransferase; IU, international unit; INR, international normalized ratio; AFP, alpha-fetoprotein.

**Table 4:** Factors associated with chronic ALBI-grade migration of the 129 BCLC-B HCC patients with acute ALBI migration after TACE.

Factors	No.	Univariate	
		OR (95% CI)	*p*
Age (years), >70/≤70	69/60	0.816 (0.392–1.697)	0.586
Gender, Male/Female	106/23	1.037 (0.402–2.675)	0.940
HBV vs. HCV ^†^	67/27	0.754 (0.287–1.981)	0.567
Alcoholic, Yes/No	18/111	1.041 (0.362–2.991)	0.940
Cirrhosis, Yes/No	85/44	0.621 (0.280–1.376)	0.241
PLT (K), ≤120/>120	35/94	1.180 (0.515–2.706)	0.695
ALT (IU/L), >40/≤40	79/50	0.635 (0.295–1.367)	0.246
AST (IU/L) ^‡^, >45/≤45	87/41	1.216 (0.558–2.648)	0.623
INR, >1.0/≤1.0	109/20	0.801 (0.285–2.253)	0.674
Tumor location, Bilobar/Unilobar	59/70	2.411 (1.123–5.176)	0.024
Tumor size			
>5 cm/≤5 cm	98/31	1.083 (0.465–2.526)	0.853
>6 cm/≤6 cm	85/44	0.999 (0.463–2.153)	0.998
>7 cm/≤7 cm	65/64	1.024 (0.494–2.120)	0.949
>8 cm/≤8 cm	51/78	1.225 (0.578–2.596)	0.596
>9 cm/≤9 cm	46/83	1.513 (0.693–3.304)	0.298
>10 cm/≤10 cm	34/95	1.338 (0.573–3.125)	0.502
>11 cm/≤11 cm	26/103	1.949 (0.720–5.277)	0.189
Tumor number			
>2 vs. ≤2	58/71	1.284 (0.615–2.682)	0.506
>3 vs. ≤3	45/84	0.906 (0.424–1.939)	0.800
AFP (ng/mL)			
>20/≤20	84/45	0.948 (0.441–2.039)	0.892
>200/≤200	57/72	0.609 (0.292–1.268)	0.185
>400/≤400	47/82	0.646 (0.306–1.366)	0.253

^†^ exclude 4 co-infection patients; ^‡^ One missing data; Abbreviations: ALBI, Albumin-Bilirubin; BCLC, Barcelona Clinic Liver Cancer; HCC, hepatocellular carcinoma; TACE, transarterial chemoembolization; OR, odds ratio; CI, confidence interval; HBV, hepatitis B virus; HCV, hepatitis C virus; PLT, platelet count; K, kilo; ALT, alanine transaminase; IU, international unit; AST, aspartate aminotransferase; INR, international normalized ratio; AFP, alpha-fetoprotein.

## Data Availability

The data presented in this study are available on request from the corresponding author. The data are not publicly available due to their massive file size.

## Supplementary Materials

The following are available online at https://www.mdpi.com/article/10.3390/cancers13174325/s1, Figure S1: Distribution of ALBI grade and rates of acute ALBI migration, Figure S2: Dynamics of ALBI score in pre-TACE phase, acute phase, and chronic phase, stratified by Seven-eleven criteria, Figure S3: Progression-free survival (PFS) and overall survival (OS) stratified by different degree of ALBI-grade migration, Table S1: The impact of NUCs on ALBI-grade migration in HBV-HCC patients undergoing TACE, Table S2: Factors associated with progression-free survival of the 531 BCLC-B HCC patients undergoing TACE, Table S3: Factors associated with overall survival of the 531 BCLC-B HCC patients undergoing TACE.
