# Supplementary material for: Effect of Transarterial Chemoembolization on ALBI Grade in Intermediate-Stage Hepatocellular Carcinoma: Criteria for Unsuitable Cases Selection
Source: Cancers (Basel). 2021 Aug 27;13(17):4325. doi: 10.3390/cancers13174325 (PMC8431519; doi:10.3390/cancers13174325)
Supplement: Supplementary file 1 [file cancers-13-04325-s001.zip › cancers-1329147-supplementary.pdf]

Supplementary Materials

Effect of Transarterial Chemoembolization on ALBI Grade in Intermediate-Stage Hepatocellular Carcinoma: Criteria for Unsuitable Cases Selection

Chen-Ta Chi, I-Cheng Lee, Rheun-Chuan Lee, Ya-Wen Hung, Chien-Wei Su, Ming-Chih Hou, Yee Chao and Yi-Hsiang Huang

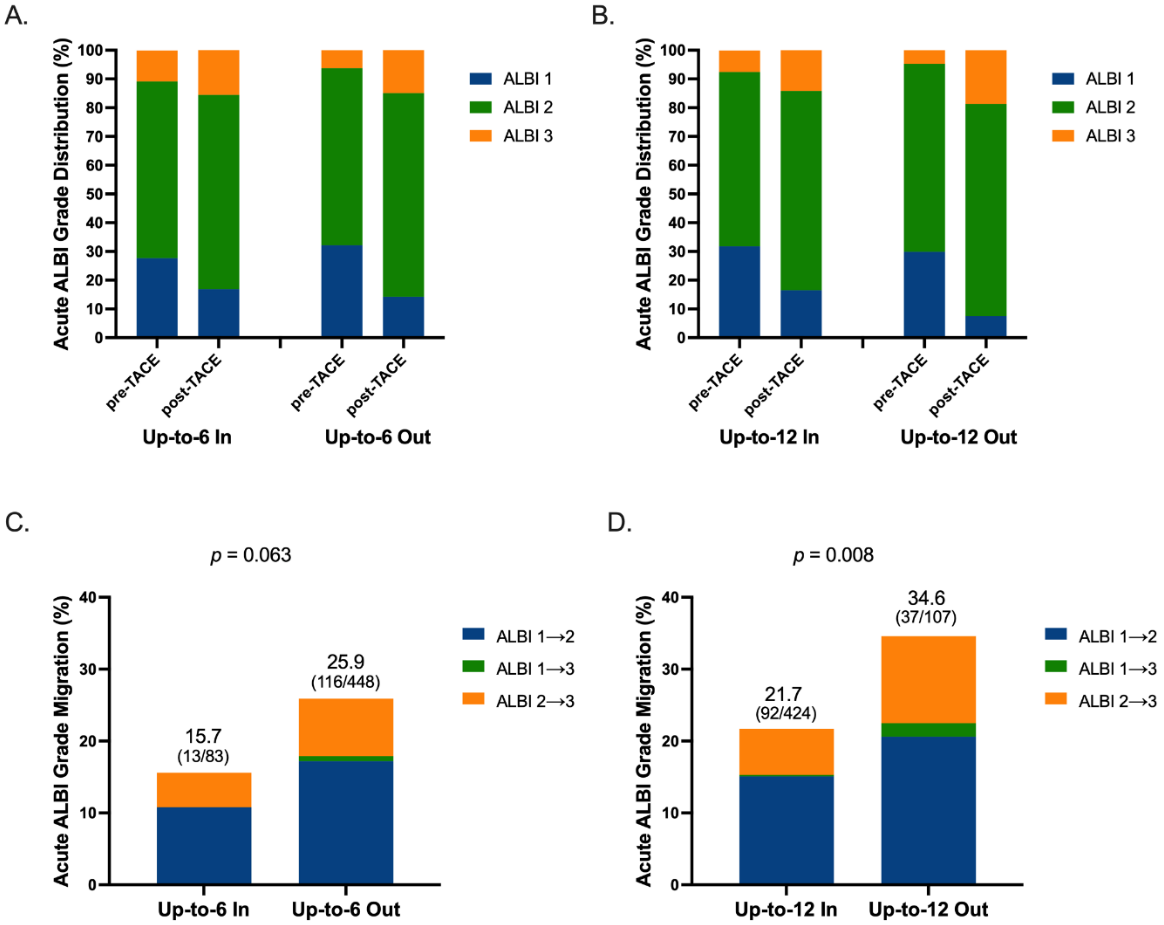

Figure S1. Distribution of ALBI grade and rates of acute ALBI migration. Distribution of ALBI grade stratified by (**A**) up-to-6 and (**B**) up-to-12, and acute ALBI grade migration rate stratified by (**C**) up-to-6 and (**D**) up-to-12. Abbreviations: ALBI, Albumin-Bilirubin; TACE, transarterial chemoembolization.

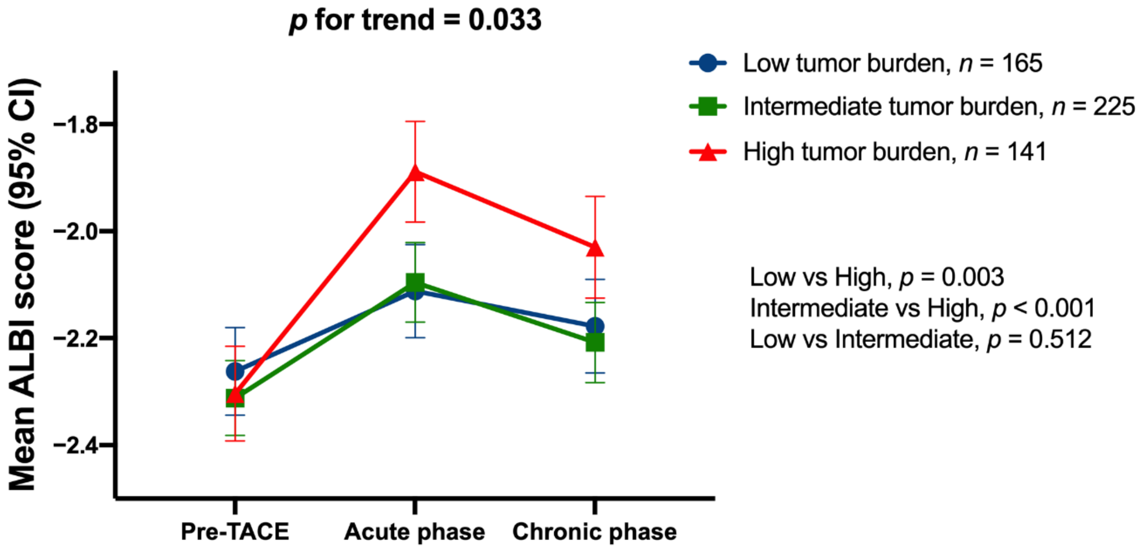

Figure S2. Dynamics of ALBI score in pre-TACE phase, acute phase, and chronic phase, stratified by seven-eleven criteria. Abbreviations: ALBI, Albumin-Bilirubin; TACE, transarterial chemoembolization; CI, confidence interval.

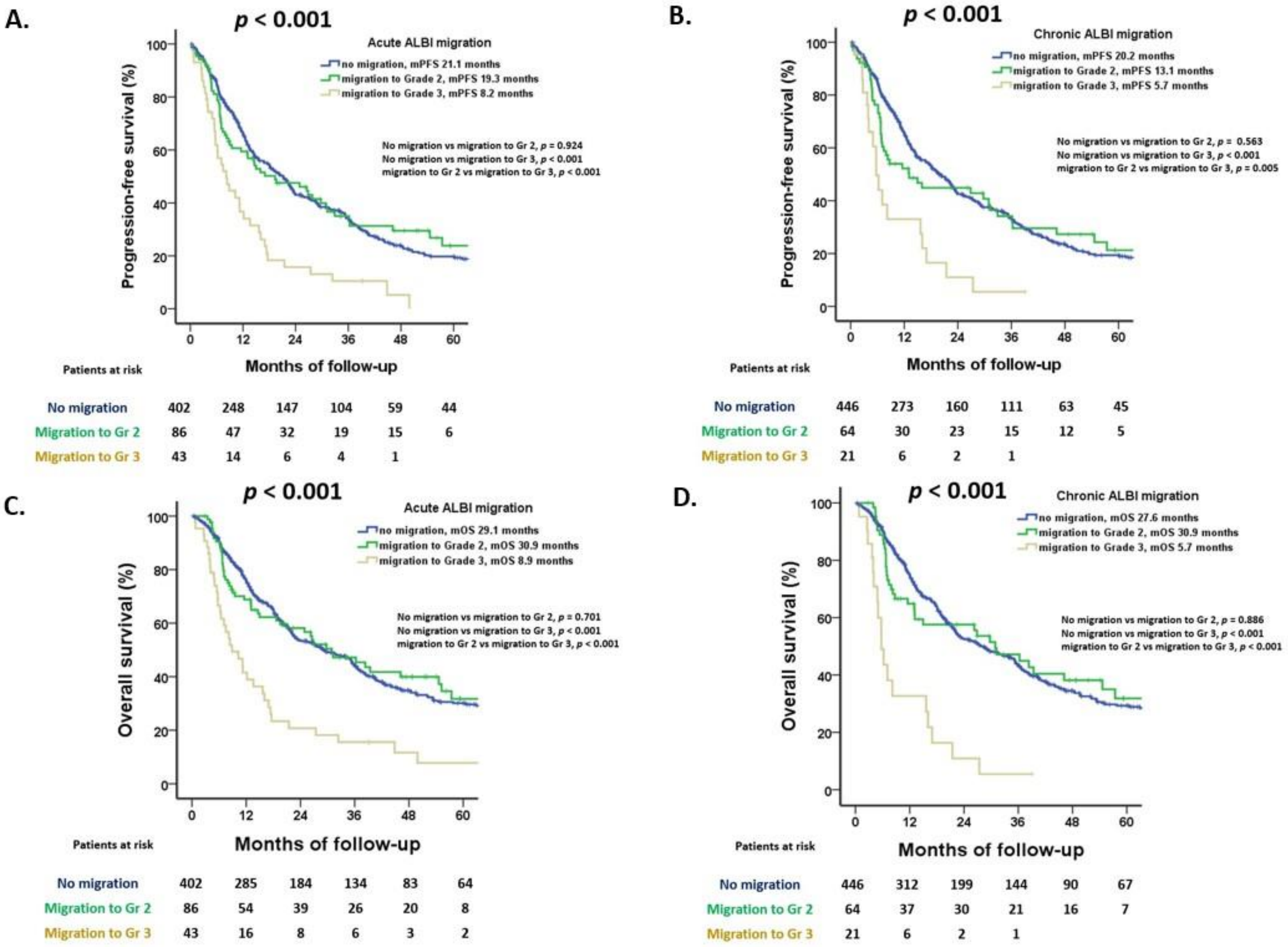

Figure S3. Progression-free survival (PFS) stratified by different degree of ALBI-grade migration, (**A**) acute ALBI-grade migration, and (**B**) chronic ALBI-grade migration. Overall survival (OS) stratified by different degree of ALBI-grade migration, (**C**) acute ALBI-grade migration, and (**D**) chronic ALBI-grade migration. Abbreviations: ALBI, Albumin-Bilirubin; mPFS, median progression-free survival; mOS, median overall survival; Gr.2, grade 2; Gr.3, grade 3.

Table S1. The impact of NUCs on ALBI-grade migration in HBV-HCC patients undergoing TACE.

Events	HBsAg Positive (*n* = 244)		*p*
	**On NUCs (*n* = 69)**	**Without NUCs (*n* = 175)**	
Acute ALBI migration, *n* (%)	22 (31.9)	49 (28.0)	0.536
Chronic ALBI migration, *n* (%)	15 (21.7)	31 (17.7)	0.472
Without NUCs (*n* = 175)	Acute ALBI grade migration		
	Yes (*n* = 49)	No (*n* = 126)	*p*
HBV reactivation, *n* (%)	3 (6.1)	5 (4.0)	0.688

Abbreviations: ALBI, Albumin-Bilirubin; HCC, hepatocellular carcinoma; TACE, transarterial chemoembolization; NUCs, nucleos(t)ide analogs; HBV, hepatitis B virus; HBsAg, hepatitis B surface antigen.

Table S2. Factors associated with progression-free survival of the 531 BCLC-B HCC patients undergoing TACE.

**Factors**	**No.**	**Univariate**		**Multivariate**			
				**Model 1**		**Model 2**	
		**HR (95% CI)**	***p***	**HR (95% CI)**	***p***	**HR (95% CI)**	***p***
Age (years), >70/≤70	265/266	1.063 (0.872–1.296)	0.546	-			
Gender, Male/Female	412/119	1.046 (0.820–1.332)	0.719	-			
HBV vs HCV †	220/156	1.163 (0.919–1.471)	0.209	-			
Alcoholic, Yes/No	66/465	1.057 (0.789–1.416)	0.709	-			
Cirrhosis, Yes/No	379/152	1.021 (0.820–1.270)	0.855	-			
PLT ≤120 K/>120 K	196/335	0.868 (0.707–1.066)	0.178	-			
ALT (IU/L), >40/≤40	306/225	0.928 (0.759–1.134)	0.465	-			
AST (IU/L) ‡, >45/≤45	325/205	1.183 (0.964–1.451)	0.109	-			
INR, >1.0/≤1.0	427/104	1.100 (0.860–1.405)	0.449	-			
Tumor location, Bilobar/Unilobar	258/273	1.033 (0.848–1.260)	0.746	-			
Tumor size, per cm	531	1.081 (1.056–1.107)	<0.001	1.071 (1.044–1.098)	<0.001	1.072 (1.045–1.099)	<0.001
Tumor number, ≥3/<3	269/262	1.078 (0.884–1.314)	0.461	-	-	-	-
AFP (ng/ml), >400/≤ 400	149/382	1.943 (1.566–2.410)	<0.001	1.753 (1.408–2.182)	<0.001	1.763 (1.418–2.193)	<0.001
Acute ALBI grade migration							
No	402	1	Ref	1	Ref	-	-
Migration to Grade 2	86	0.985 (0.743–1.305)	0.916	0.865 (0.650–1.150)	0.318	-	-
Migration to Grade 3	43	2.272 (1.616–3.196)	< 0.001	1.637 (1.150–2.329)	0.006	-	-
Chronic ALBI grade migration							
No	446	1	Ref	-	-	1	Ref
Migration to Grade 2	64	1.094 (0.803–1.490)	0.569	-	-	0.948 (0.692–1.300)	0.742
Migration to Grade 3	21	2.733 (1.694–4.410)	< 0.001	-	-	2.029 (1.247–3.302)	0.004

† exclude 24 co-infection patients; ‡ 1 missing data; Abbreviations: ALBI, Albumin-Bilirubin; BCLC, Barcelona Clinic Liver Cancer; HCC, hepatocellular carcinoma; TACE, transarterial chemoembolization; HR, hazard ratio; CI, confidence interval; HBV, hepatitis B virus; HCV, hepatitis C virus; PLT, platelet count; K, kilo; ALT, alanine transaminase; AST, aspartate aminotransferase; IU, international unit; INR, international normalized ratio; AFP, alpha-fetoprotein

Table S3. Factors associated with overall survival of the 531 BCLC-B HCC patients undergoing TACE.

Factors	No.	Univariate		Multivariate			
				Model 1		Model 2	
		HR (95% CI)	*p*	HR (95% CI)	*p*	HR (95% CI)	*p*
Age (years), >70/≤70	265/266	1.230 (0.994–1.520)	0.056	1.412 (1.134–1.757)	0.002	1.445 (1.160–1.800)	0.001
Gender, Male/Female	412/119	1.114 (0.858–1.446)	0.418	-	-	-	-
HBV vs HCV[†]	220/156	1.032 (0.803–1.327)	0.804	-	-	-	-
Alcoholic, Yes/No	66/465	1.147 (0.846–1.557)	0.377	-	-	-	-
Cirrhosis, Yes/No	379/152	1.052 (0.834–1.328)	0.667	-	-	-	-
PLT, ≤120 K/>120 K	196/335	0.910 (0.731–1.132)	0.398	-	-	-	-
ALT (IU/L) >40/≤40	306/225	1.035 (0.834–1.283)	0.757	-	-	-	-
AST (IU/L) [‡] >45/≤45	325/205	1.332 (1.067–1.662)	0.011	1.256 (0.995–1.586)	0.055	1.289 (1.025–1.622)	0.030
INR, >1.0/≤1.0	427/104	1.102 (0.848–1.430)	0.467	-	-	-	-
Tumor location, Bilobar/Unilobar	258/273	1.012 (0.819–1.250)	0.913	-	-	-	-
Tumor size, per cm	531	1.090 (1.063–1.118)	<0.001	1.083 (1.053–1.113)	<0.001	1.084 (1.055–1.114)	<0.001
Tumor number, ≥3/<3	269/262	1.119 (0.905–1.383)	0.298	-	-	-	-
AFP (ng/ml) >400/≤ 400	149/382	1.985 (1.583–2.489)	<0.001	1.826 (1.448–2.303)	<0.001	1.825 (1.449–2.297)	<0.001
Acute ALBI grade migration							
No	402	1	Ref	1	Ref	-	-
Migration to Grade 2	86	0.941 (0.693–1.277)	0.697	0.784 (0.573–1.072)	0.128	-	-
Migration to Grade 3	43	2.451 (1.726–3.481)	< 0.001	1.551 (1.070–2.251)	0.021	-	-
Chronic ALBI grade migration							
No	446	1	Ref	-	-	1	Ref
Migration to Grade 2	64	0.970 (0.691–1.360)	0.858	-	-	0.791 (0.552–1.106)	0.164
Migration to Grade 3	21	3.899 (2.407–6.315)	< 0.001	-	-	2.785 (1.697–4.570)	< 0.001

[†] exclude 24 co-infection patients; [‡] 1 missing data; Abbreviations: ALBI, Albumin-Bilirubin; BCLC, Barcelona Clinic Liver Cancer; HCC, hepatocellular carcinoma; TACE, transarterial chemoembolization; HR, hazard ratio; CI, confidence interval; HBV, hepatitis B virus; HCV, hepatitis C virus; PLT, platelet count; K, kilo; ALT, alanine transaminase; AST, aspartate aminotransferase; IU, international unit; INR, international normalized ratio; AFP, alpha-fetoprotein

© 2021 by the authors. Licensee MDPI, Basel, Switzerland. This article is an open access article distributed under the terms and conditions of the Creative Commons Attribution (CC BY) license (http://creativecommons.org/licenses/by/4.0/).
